# Enhanced Reliability of a-IGZO TFTs with a Reduced Feature Size and a Clean Etch-Stopper Layer Structure

**DOI:** 10.1186/s11671-019-3001-3

**Published:** 2019-05-16

**Authors:** Jae-Moon Chung, Fang Wu, Seung-Woo Jeong, Ji-Hoon Kim, Yong Xiang

**Affiliations:** 1 School of Materials and Energy, University of Electronic Science and Technology of China, 2006 Xiyuan Avenue, West High-Tech Zone, Chengdu, 611731 Sichuan China; 2 Chongqing BOE Optoelectronics Technology Co., Ltd, Chongqing, 400718 China; 3 BOE Technology Group Co., Ltd, Beijing, 100176 China

**Keywords:** Gate drive IC on array (GOA), Thin-film transistors (TFTs), a-IGZO, Back channel etch, Etch stopper layer

## Abstract

The effects of diffuse Cu^+^ in amorphous indium-gallium-zinc-oxide (a-IGZO) thin-film transistors (TFTs) on the microstructure and performance during a clean etch stopper (CL-ES) process and a back channel etch (BCE) process are investigated and compared. The CL-ES layer formed with a clean component, as verified by TOF-SIMS, can protect the a-IGZO layer from the S/D etchant and prevent Cu^+^ diffusion, which helps reduce the number of accepter-like defects and improve the reliability of the TFTs. The fabricated CL-ES-structured TFTs have a superior output stability (final *I*_ds_/initial *I*_ds_ = 82.2 %) compared to that of the BCE-structured TFTs (53.5%) because they have a better initial SS value (0.09 V/dec vs 0.46 V/dec), and a better final SS value (0.16 V/dec vs 0.24 V/dec) after the high current stress (HCS) evaluation. In particular, the variation in the threshold voltages has a large difference (3.5 V for the CL-ES TFTs and 7.2 V for the BCE TFTs), which means that the CL-ES-structured TFTs have a higher reliability than the BCE-structured TFTs. Therefore, the CL-ES process is expected to promote the widespread application of a-IGZO technology in the semiconductor industry.

## Background

Recently, display products have emphasized not only large areas and high resolutions, but also aesthetically pleasing exterior designs [1–3]. Narrow bezels have been adopted as one of the vital features for this design emphasis [4]. To realize this, it is essential to integrate the main circuits that drive the display into the panel. Gate drive IC on array (GOA) is a relatively simple and commonly used method, where the gate signal enters the panel one line after another and the *V*_*on*_ moves sequentially each time. GOA has multiple advantages, such as decreased cost (elimination of the G-IC costs, removal of the G-IC bonding process, increased glass substrate utilization, etc.) and aesthetic effect (narrow bezels or borderless devices) [5]. Unlike individual pixel TFTs, however, GOA TFTs require more stringent reliability conditions to achieve a higher output current and longer on-time performance. With the recently increasing market demands for high-resolution products, reliability enhancement of the GOA performance has become urgent and necessary [6].

Amorphous indium-gallium-zinc-oxide (a-IGZO) is widely used in the display industry due to its high saturation electron mobility (5~10 cm^2^/V s) and low off-current (< 1 pA) [7, 8]. The back channel etch (BCE) technology is commonly used for the production of TFTs in industry [9, 10]. BCE-structured a-IGZO TFTs have satisfactory characteristics for individual pixel TFTs and the size reduction of GOA TFTs. However, some key TFT characteristics, particularly the output current stability, cannot satisfy the high current stress (HCS) environment required for GOA TFTs [11–13], mainly due to two features of the BCE process [14]. The first is that the surface of an a-IGZO film (back channel of a-IGZO TFT) is exposed to S/D etchants, which traditionally consist of HNO_3_, H_3_PO_4_, and CH_3_COOH, that have a fast etching rate that is not controllable for a-IGZO films [15]. A mild H_2_O_2_-based etchant with stable etching and minimal damage to the a-IGZO films may be used for the S/D electrode (Cu metal) etching, but damage to the surface of the a-IGZO film is still inevitable [16]. Second, the direct contact of the S/D metal (Mo/Cu/Mo) with the a-IGZO film may contaminate the TFT back channel [17]. Fortunately, a clean etch stopper (CL-ES) process, which is less complicated and costly and minimizes contamination, can be used to fabricate a-IGZO-based TFTs with improved uniformity and stability for large-area displays [18]. Although the CL-ES-structured TFT shows an improved performance, the questions of how the etchant will react with a-IGZO film and how Cu^+^ diffusion into a-IGZO films affects the microstructure and performance of the devices remain unclear.

In this study, a-IGZO GOA TFTs with a reduced feature size and clean back channel structure were fabricated via a CL-ES process by batch etching of multilayer a-IGZO/Mo/Cu/Mo. Moreover, the influence of the etchant and Cu^+^ diffusion on the microstructure and performance of CL-ES-structured a-IGZO GOA TFT devices are studied and compared with those of BCE-structured a-IGZO GOA TFT devices. More importantly, the etch stopper layer of the CL-ES device serves as the S/D etchant protection layer as well as the Cu^+^ diffusion barrier layer, which helps to reduce the amount of defects and improve the reliability of the high current stress reliability, SS values, high current stress and threshold voltage variations, etc. Therefore, this work provides direct evidence and an insightful demonstration that the improved performance of CL-ES-structured TFTs is highly correlated with its CL-ES structure and its clean components and confirms that the CL-ES process might be an efficient route for the mass production of displays with satisfactory performances.

## Experimental Methods

### Fabrication of a-IGZO GOA TFTs

**Fig. 1:**
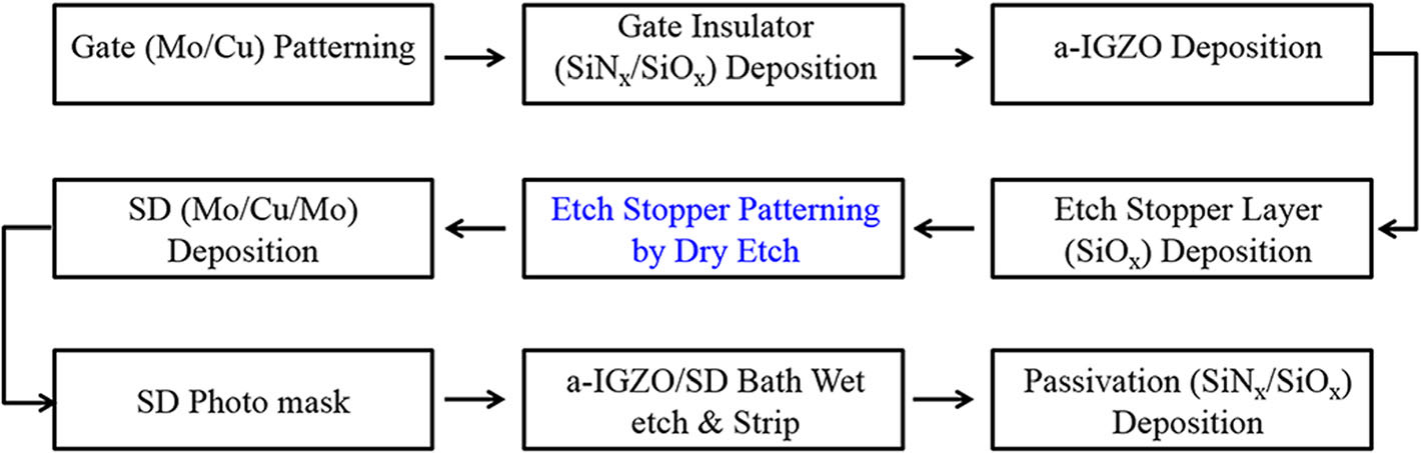
Fabrication process of the a-IGZO GOA TFT

The CL-ES-structured a-IGZO TFT devices were fabricated via a modified five-step CL-ES process (Fig. 1), as reported in our previous work [15]. First, the gate electrode was formed with Mo/Cu metal and the gate insulator was deposited with a SiNx/SiOx (3000 Å/1000 Å) double layer using PECVD at 340 °C. Second, an a-IGZO film of 300 Å was deposited using DC magnetron reactive sputtering at room temperature with a partial pressure of oxygen of 15%. An etch stopper layer (SiOx, ESL) of 1000 Å was deposited using PECVD at 240 °C and reactively etched by CF_4_ plasma for patterning, using the active photolithography mask of the BCE process as the etch mask. For this step, the a-IGZO film under the ES layer patterns was protected from exposure to the CF_4_ plasma, while the rest of the a-IGZO film, not protected by the ES layer patterns, was not etched either but was converted into a conductive film. Third, the source-drain (S/D) electrodes (Mo/Cu/Mo triple layers) were sputter-deposited and etched using an H_2_O_2_ etchant containing 0.2 wt% of a fluoride additive, with the S/D photolithography mask and the ES layer pattern serving as the etch mask. Fourth, a passivation layer of 3000 Å was deposited. The subsequent processes were similar to those of a typical TFT LCD backplane fabrication.

For comparison, BCE-structured a-IGZO TFT devices were fabricated using the conventional BCE process and the same BCE mask.

### Characterization

The morphologies, microstructures, and compositions of the samples were characterized using SEM (Camscan Mx2600FE), X-ray photoelectron spectroscopy (XPS, PHI Quantera II), and time-of-flight secondary ion mass spectrometry (IONTOF, TOF-SIMS 5). Electric measurements were carried out using a semiconductor characteristic analyzer (Keysight 4082A) in a dark environment and at 60 °C. For simplicity, the HCS reliability was evaluated for over 1000 s with *V*_gs_ at 25 V and *V*_ds_ at 25 V. During the evaluation, the state of the GOA TFT was monitored by measuring the *I*_ds_ current at 1-s intervals, and the trend of the *I*_ds_ current was analyzed. The *I*_*d*_*-V*_*g*_ transfer characteristics were also monitored at 100-s intervals.

## Results and Discussion

**Fig. 2:**
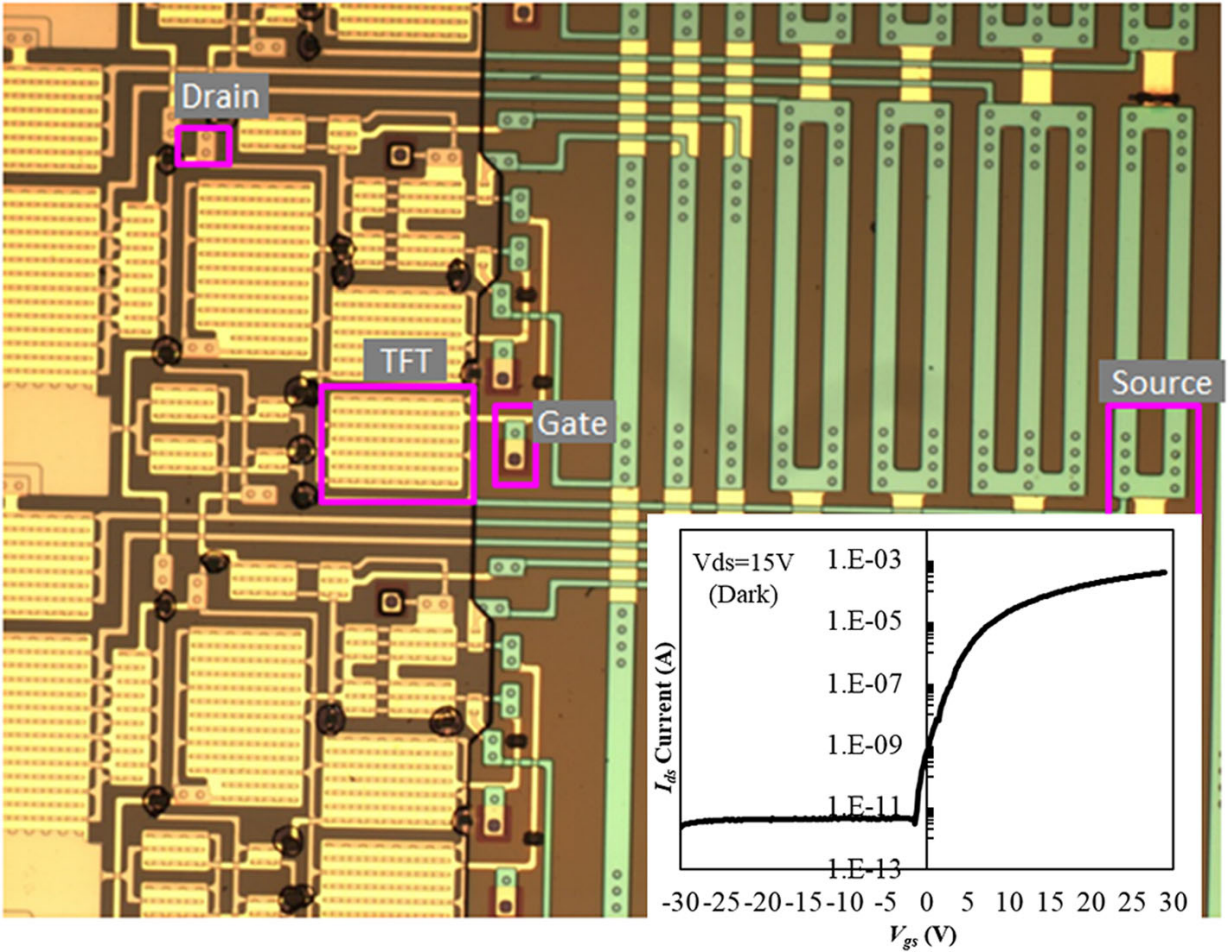
Photographs of the GOA TFTs electrically separated from the GOA circuits (insert: *I*_off_ noise current between the TFT of interest and the other peripheral TFTs)

The GOA TFT device, containing TFT channels and gate, drain, and source components, as manufactured by the CL-ES process, is shown in Fig. 2. To accurately measure each TFT characteristic, all the TFTs were disconnected using a laser, thus becoming independent, so that the gate, source, and drain could not share a node with any other TFT. As marked by the red line in Fig. 2, this TFT has a multichannel and separated GOA structural design, with a channel width and length of 120 μm and 10 μm, respectively, for convenience of the electrical measurements. This TFT is also designed to have an average level of current flow to the individual TFT channels by placing a floating piece of metal (located in the middle of the channels), which integrates each channel. Before the HCS reliability evaluation, the separated operation reliability is confirmed first by evaluating the electrical interference of the TFT of interest from the other peripheral TFTs. In this case, the *I*_off_ noise current of the separated GOA TFTs is measured to be 3 pA (insert curve in Fig. 2), confirming that there is no electrical interference from the other GOA constituent devices in the vicinity.

**Fig. 3:**
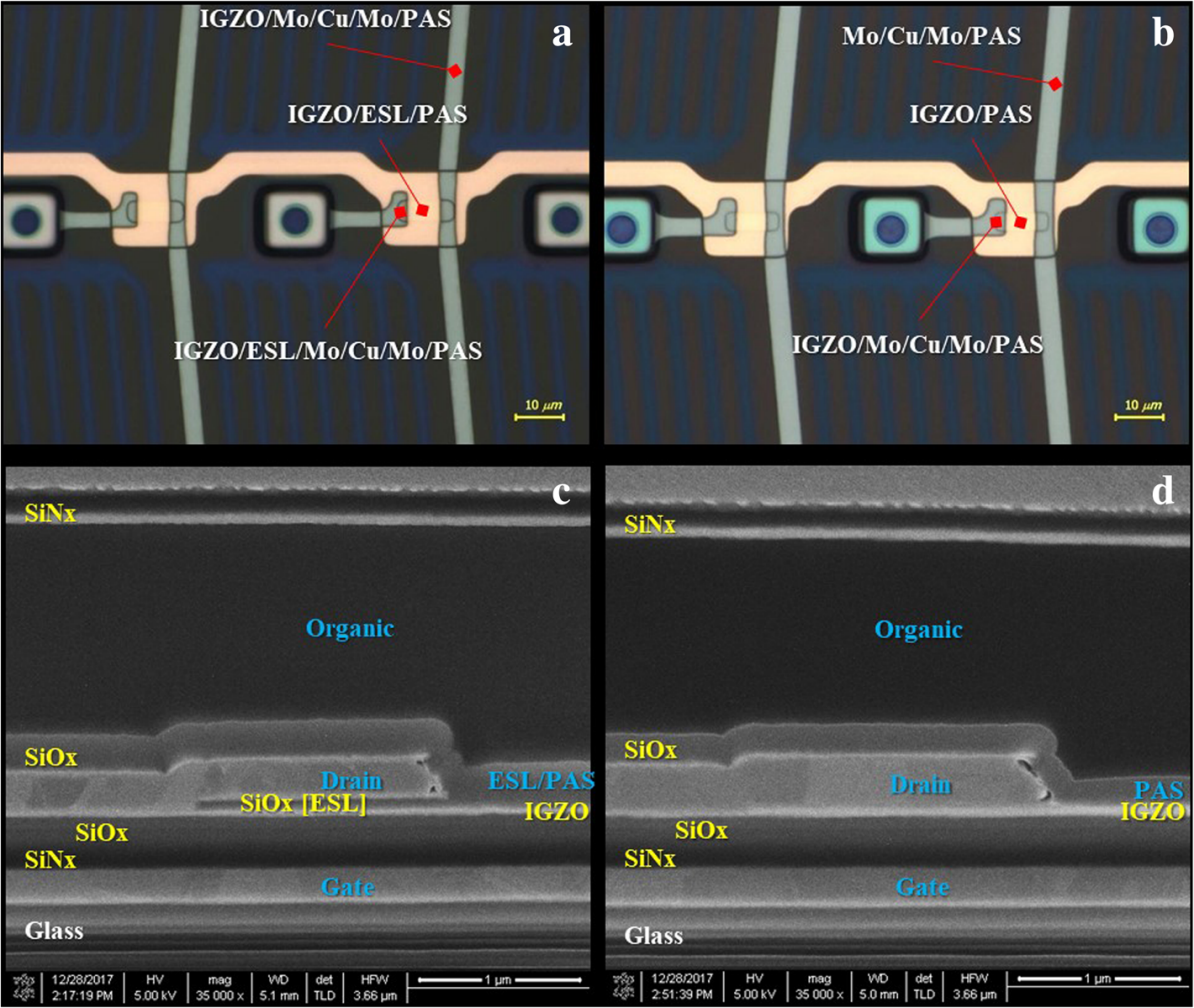
SEM images of the a-IGZO TFTs: **a** CL-ES-structured TFT top view, **b** BCE-structured TFT top view, **c** CL-ES-structured TFT cross-sectional view, and **d** BCE-structured TFT cross-sectional view

Several feature sizes of the CL-ES-structured TFTs and BCE-structured TFTs are measured and compared. For the CL-ES-structured TFTs (Fig. 3a), the width and length are 4 μm and 6 μm, respectively, similar to those of the BCE-structured a-IGZO TFTs in Fig. 3b. Generally, the BCE process is desirable for oxide TFT manufacturing due to its small feature size. Therefore, the obtained CL-ES-structured TFTs show a decreased feature size and an integration degree as high as the BCE-structured TFTs. Moreover, the cross-sectional size of the CL-ES-structured TFTs is similar to that of the BCE-structured TFTs (Fig. 3c, d), while the CL-ES-structured TFTs show a distinct ES layer that is not observed in the BCE TFTs. The CL-ES process primarily forms ES patterns, while the batch etching process on multilayered a-IGZO/Mo/Cu/Mo can be carried out with similar masks for the active patterns and source-drain electrodes as those in the BCE process. Therefore, except for the ES patterns, the number of photolithographic masks used in the CL-ES process is the same as for the BCE process. This CL-ES process can avoid the increased number of masks of the conventional ESL process and has a reduced feature size, making it economically viable for mass production. In addition, without using the half tone exposure, a process simplification procedure conventionally used in the TFT LCD industry, the process complication and the manufacturing cost are both reduced.

**Fig. 4:**
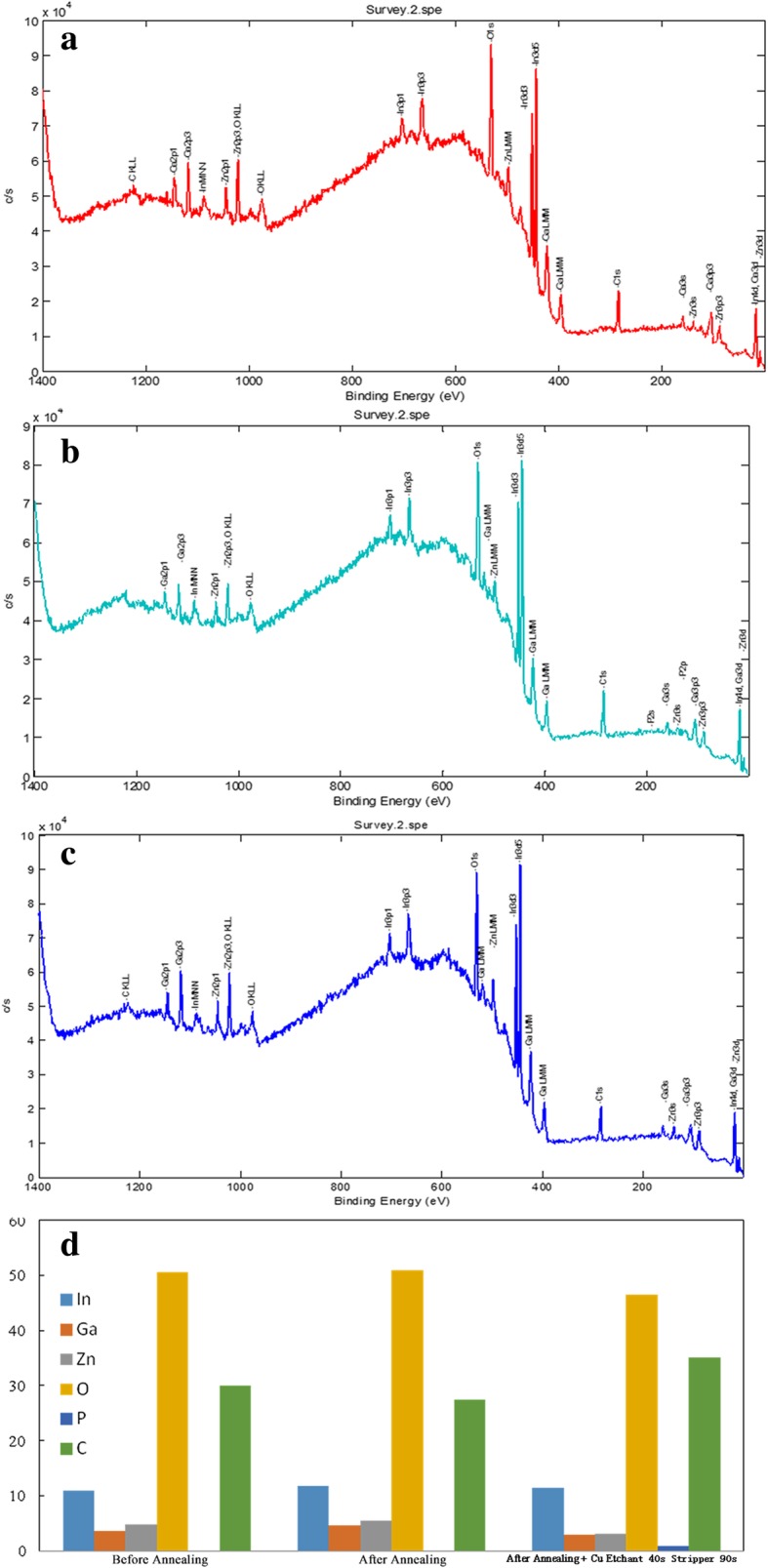
XPS analysis of the surface composition of the a-IGZO thin films **a** before annealing, **b** after annealing, and **c** after exposure to the H_2_O_2_ Cu etchant during the BCE process. **d** Corresponding atomic percentages for the above process

To further observe the surface defects of the BCE-structured TFTs during the BCE fabrication process, the surface composition of a-IGZO films before annealing (sample 1), after annealing (sample 2), and after exposure to the H_2_O_2_ Cu etchant (sample 3) is studied via XPS. In the fully scanned spectra of a-IGZO films (Fig. 4a–c), peaks for In, Ga, Zn, O, and C elements exist during the BCE fabrication process. As shown in Fig. 4d, although the BCE-structured TFT shows no significant change in the composition of the a-IGZO films before annealing (sample 1) and after annealing at 330 °C for 1 h (sample 2), significant changes are observed after exposure to wet chemicals (sample 3). In particular, zinc, which has a relatively low binding energy with oxygen, is found to be 4.82% in sample 1 and 5.42% in sample 2, but it has decreased to 3.16% in sample 3. Indium has minimal variation in the compositions among the different processes, and the relative percentage change of Zn with respect to In is tremendous, namely, 44.1%, 46.0%, and 27.6% for samples 1, 2, and 3, respectively. This is similar for gallium, which also has a strong binding affinity with oxygen. In other words, during the wet etching process, undesired defects, including a substantial loss of Zn and Ga, occurred on the exposed back surface of the oxide semiconductor. The reasons for this phenomenon may be related to their different binding energies to oxygen and the different molecular structures of the a-IGZO film [19].

It is well known that the chemical resistance of a-IGZO films to acidic etchants is very weak [20]. In particular, the abrupt loss of Zn, which is believed to determine the molecular structure of a-IGZO, causes a weakening of the surface structure of the a-IGZO films. In addition, the reduction of Ga, which suppresses carrier generation via its strong binding energy with oxygen, may increase the probability of developing oxygen vacancies [Vo] [21]. Therefore, BCE-structured GOA TFTs cannot avoid etching damage to the TFT back channel, even in a relatively mild H_2_O_2_-based Cu etchant.

**Fig. 5:**
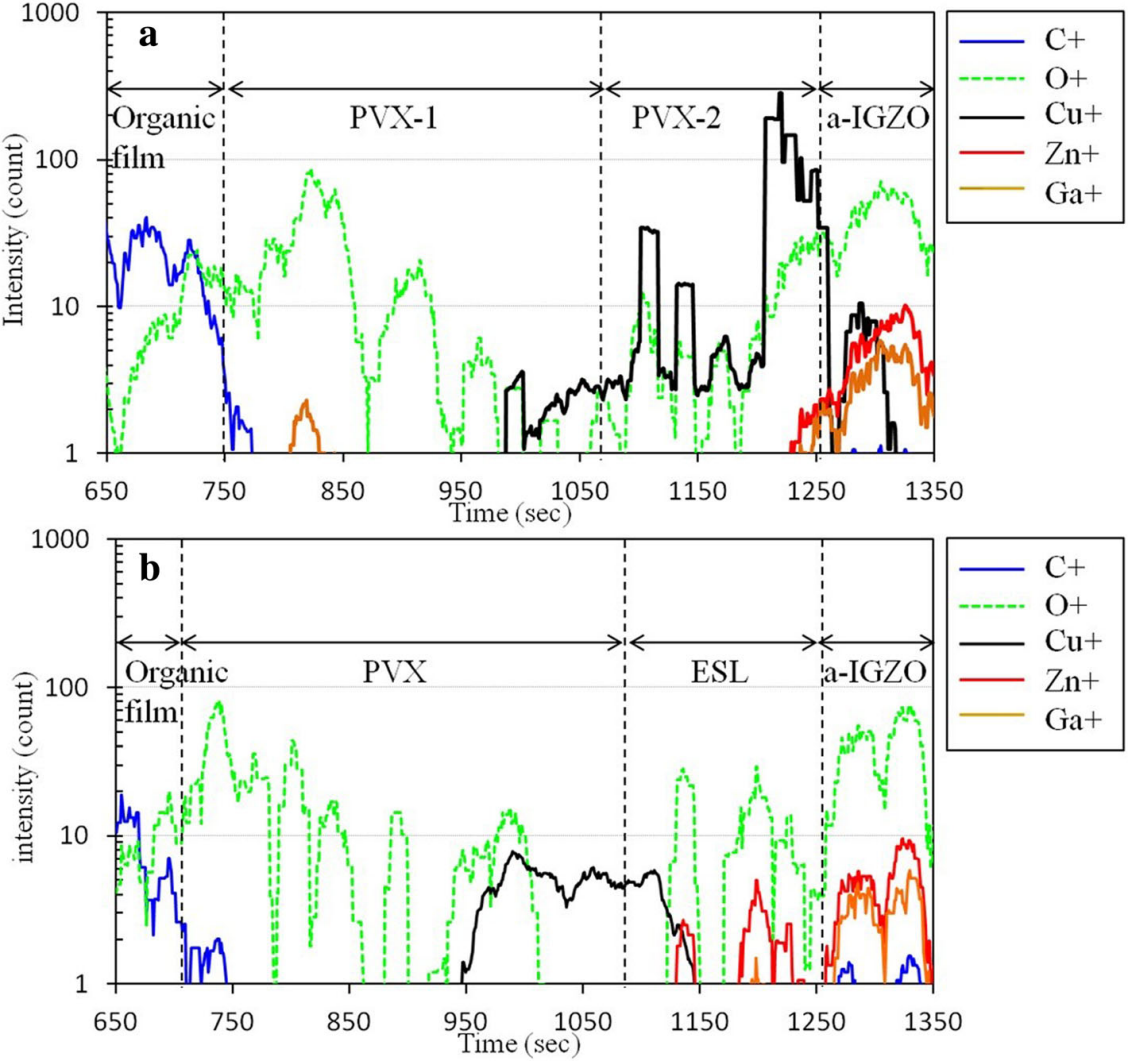
TOF-SIMS analysis of the channel regions of a-IGZO TFTs fabricated via **a** the BCE and **b** the CL-ES processes

To confirm the protection of the ES layer, the composition of the a-IGZO TFT channel region is studied by using TOF-SIMS for samples prepared by the BCE and CL-ES (clean etch stopper) processes (Fig. 5). Since Cu^+^ in the a-IGZO film can produce accepter-type defects and trap electrons, the a-IGZO TFT channel must be clean to enhance the electrical stability. As observed, the Cu^+^ peak detected in the BCE sample is 20 times greater than that of the CL-ES sample. Moreover, the detection region of Cu^+^ overlaps with the detection region of Zn^+^ and Ga^+^ to a great extent (Fig. 5a). These results indicated that the a-IGZO films in the BCE-structured TFTs are contaminated by Cu^+^ due to the direct contact of the a-IGZO film in the TFT back channel region with the Cu metal. For the CL-ES-structured TFTs (Fig. 5b), Cu^+^ is only detected in the ES region, indicating that direct contact of the a-IGZO TFT channel region with the Cu metal is avoided. Surprisingly, a considerable amount of Zn^+^ appears in the ESL. The diffused Zn^+^ is caused by the higher pretreatment plasma conditions and pressure conditions during ESL deposition. Therefore, the ES layer in CL-ES-structured TFTs is essential to improve the electrical stability by avoiding surface damage to and contamination of the a-IGZO films.

**Fig. 6:**
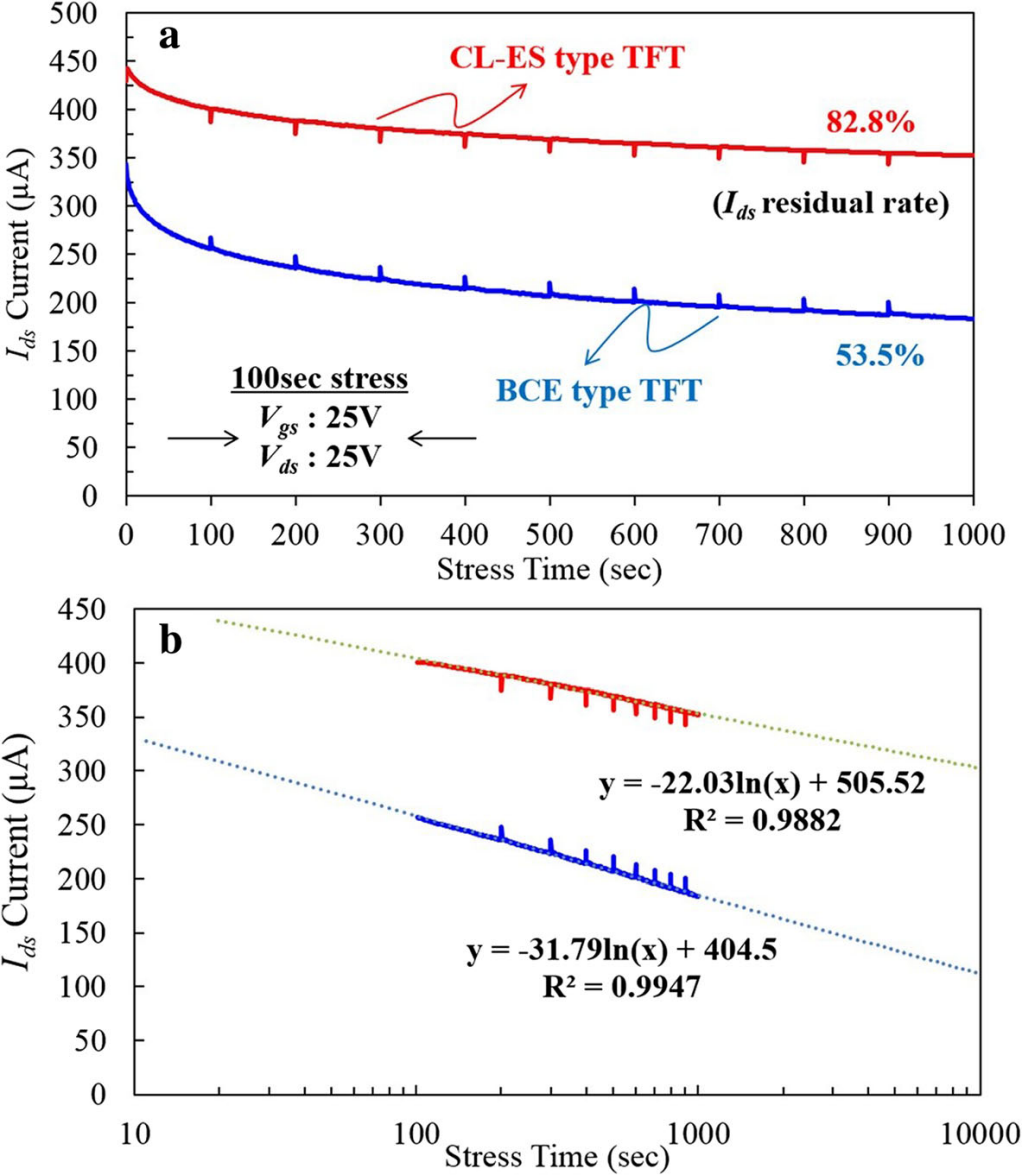
**a** Experimental data for 1000 s and **b** extrapolation for long-term HCS evaluation of the CL-ES- and BCE-structured GOA TFTs

The high current stress (HCS) evaluation for the CL-ES- and BCE-structured GOA a-IGZO TFTs is shown in Fig. 6a. For the same feature sizes, the initial *I*_ds_ current of the CL-ES-structured TFT is 429 μA, which is higher than that of the BCE-structured TFT (343 μA). After the HCS evaluation for 1000 s, the *I*_ds_ current of the CL-ES-structured TFT is 352 μA, approximately 82.2 % of its initial value. In contrast, the *I*_ds_ residual current of the BCE-structured TFT has decreased to 183 μA and only maintains 53.5% of its initial value. Furthermore, as evaluated by extrapolation (Fig. 6b), the *I*_ds_ residual current of the CL-ES-structured TFT is expected to be 302.6 μA, maintaining 70.5% of its initial value after 10,000 s. For the BCE-structured TFT, the *I*_ds_ residual current sharply decreases to 111.7 μA, maintaining only 33.7% of its initial value. Therefore, under the same output characteristics, the degree of integration for the GOA TFT fabricated via the CL-ES process can be increased by as much as 271% compared to that of the BCE process.

**Fig. 7:**
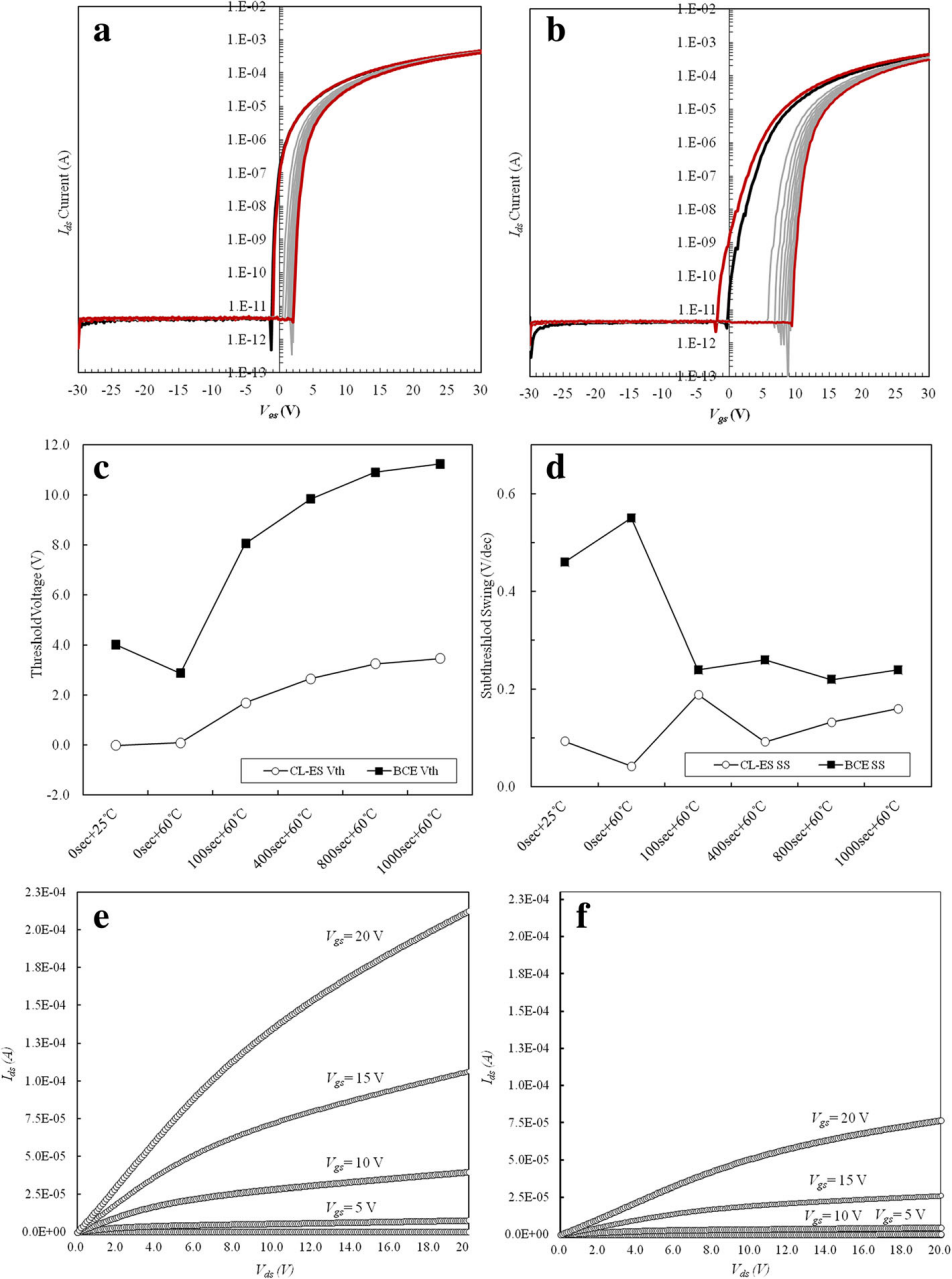
*I*-*V* transfer characteristics measured during the HCS evaluation of **a** the CL-ES and **b** the BCE-structured GOA TFTs. Behavior of **c** the threshold voltage and **d** the sub-threshold swing at intervals of 1000 s and *V*_ds_ = 15 V. The initial measurements of the *I*_*d*_*-V*_*d*_ output characteristics for **e** the CL-ES- and **f** the BCE-structured GOA TFTs with *V*_gs_ = 0, 5, 10, 15, and 20 V

Additionally, the *I*-*V* transfer characteristics of both CL-ES- and BCE-structured GOA TFTs during the HCS reliability evaluation are also measured (Fig. 7 and Table 1). For CL-ES-structured TFT (Fig. 7a), the threshold voltage is 0.0 V in the initial HCS evaluation (25 °C) and 3.5 V after the HCS evaluation at 60 °C for 1000 s. Moreover, the threshold voltage continuously shifts in the positive direction with a total change (*ΔV*_th_) of 3.5 V. The sub-threshold swing (SS) value is slightly increased from 0.09 to 0.16 V/dec. For the BCE-structured TFT, the threshold voltage is much higher, namely, 4.0 V at 25 °C, and increases to 11.2 V after HCS evaluation at 60 °C for 1000 s. A possible reason for these high threshold voltages is the diffusion of Cu^+^ into the a-IGZO film during the wet etching process of the BCE process. Cu^+^ can act as accepter-type defect sites in a-IGZO films, and a high density of Cu^+^ can trap a large number of electrons. The trapped electrons generate a screened coulombic potential that results in the transient threshold voltage shift phenomenon. Generally, the gate insulator bulk and the newly formed defect sites inside the bulk of the a-IGZO films can increase the SS value of TFTs [11]. These results clearly explain the decrease of the *I*_ds_ residual current in the BCE-structured TFTs. However, the SS value of the BCE-structured TFT shows a tendency to decrease from 0.46 to 0.24 V/dec. This decreasing SS value results from electrons accumulating near the a-IGZO interface, after which the gate insulator can rapidly fill up the high levels of the accepter-type trap sites that existed initially. Moreover, the trap sites are filled faster than they are generated by the HCS, and therefore, the number of trapped electrons gradually decreases over time. This agrees with the positively shifting behavior of the threshold voltage.

**Table 1:** *I*-*V* transfer characteristics measured during the HCS evaluation of the CL-ES- and the BCE-structured GOA TFTs

Test conditions		CL-ES-structured GOA TFT				BCE-structured GOA TFT			
Time (s)	Temp (^o^C)	*I*_on_ (μA)	*V*_th_ (V)	Mobility (cm^2^/V s)	SS (V/dec)	*I*_on_ (μA)	*V*_th_ (V)	Mobility (cm^2^/V s)	SS (V/dec)
0	25	133.1	0.0	7.2	0.09	61.9	4.0	9.1	0.46
0	60	136.4	0.1	7.3	0.04	76.5	2.9	9.5	0.55
100	60	113.5	1.7	7.6	0.19	34.4	8.1	11.0	0.24
400	60	100.4	2.7	7.7	0.09	21.1	9.9	11.3	0.26
700	60	92.1	3.3	7.9	0.13	15.7	10.7	11.5	0.22
1000	60	89.5	3.5	7.9	0.16	12.5	11.2	11.6	0.24

As for the uniformity of the characteristics for CL-ES, because ESL provides active back channel protection from Cu^+^ contamination and etchant damage, its result is stable compared to that of BCE. In addition, it should be noted that the characteristics of the output curve show no differences for BCE and can promise CL-ES production and stability (Table 2, Fig. 7e, f).

**Table 2:** Five locations of initial *I*-*V* transfer characteristics for the CL-ES- and the BCE-structured GOA TFTs

No.	CL-ES-structured GOA TFT				BCE-structured GOA TFT			
	*I*_on_ (μA)	*V*_th_ (V)	Mobility (cm^2^/V s)	SS (V/dec)	*I*_on_ (μA)	*V*_th_ (V)	Mobility (cm^2^/V s)	SS (V/dec)
#1	133.1	0	7.2	0.09	61.9	4	9.1	0.46
#2	138.6	− 0.4	7.4	0.08	72.8	3.2	9.3	0.42
#3	128.2	0.4	7.1	0.13	74.2	3.8	9.4	0.42
#4	135.8	− 0.4	7.3	0.14	66.6	4.2	9	0.49
#5	122.8	0.5	7.2	0.12	57.5	4.8	9	0.51
Average values	131.7	0.02	7.24	0.112	66.6	4	9.16	0.46
Standard deviations	6.2817	0.4266	0.1020	0.0259	7.0905	0.5215	0.1817	0.0406

Figure 7 c and d show the results of the sub-threshold swing and threshold voltage behavior along with the HCS evaluation progress. Generally, the sub-threshold swing value of the GOA TFT gradually increases, as seen for the CL-ES-structured TFT (Fig. 7d). However, the BCE-structured TFT shows abnormal behavior, with the sub-threshold swing value increasing initially and subsequently decreasing during the HCS evaluation. The SS value of the BCE-structured TFT increases from 0.46 to 0.55 V/dec when the substrate temperature increased from 25 to 60 °C. At the same time, the threshold voltage negatively shifts from 4.0 to 2.9 V (Fig. 7c). This abnormal phenomenon results from the damage of the a-IGZO film surface by the H_2_O_2_ etchant with added fluoride. As mentioned before, the surface damage of the a-IGZO films implies a lack of Zn, Ga, and oxygen atoms, which forms numerous defect sites, including oxygen vacancies. It is believed that these defect sites are active as shallow-donor-like states, which are close to the minimum conduction band, and are capable of thermal excitation and acting as electron sources for the conduction band, leading to a degradation of the a-IGZO TFT characteristics. Based on the above results, the CL-ES-structured TFT with small-accepter-like states and oxygen deficiencies that act as shallow-donor-like states is a much better structure than the BCE-structured TFT.

## Conclusion

In conclusion, we demonstrate that CL-ES-structured GOA TFT, with a decreased device feature size and a clean etch stopper layer, can significantly improve the device performance and stability. With the proposed CL-ES-structured TFT manufacturing process, the damage and contamination of the TFT back channel are minimized. In addition, for the same degree of integration as that of the BCE-structured GOA TFT, the CL-ES-structured TFT process can meet the goals of aesthetic design and manufacturing cost efficiency. The CL-ES-structured GOA TFT shows excellent electrical performance compared to that of the BCE-structured GOA TFT, including a much higher residual ion current (~ 187%), much lower initial SS value (0.09 V/dec), and a much lower variation of the threshold voltage (3.5 V). This implies the possibility of GOA designs with much higher integration and reliability. The enhanced performance and stability suggest that the CL-ES-structured TFT, with a simplified process and a clean etch stopper layer, successfully overcomes the donor-like defects caused by oxygen deficiencies and the accepter-like defects caused by Cu^+^ diffusion during the BCE process. Therefore, a clean surface composition for the a-IGZO channel region in CL-ES-structured TFTs is important for the production of a-IGZO TFT backplanes with high-reliability, high-resolution, and narrow-bezel displays.

## Abbreviations

TFT: Thin-film transistor
GOA: Gate drive IC on array
a-IGZO: Amorphous indium-gallium-zinc-oxide
LCD: Liquid crystal display
PEVCD: Plasma-enhanced chemical vapor deposition
ESL: Etch stopper layer
BCE: Back channel etch
HCS: High current stress
SiOx: Silicon oxide
SiNx: Silicon nitride
SS: Sub-threshold swing
